# Halloween genes in panarthropods and the evolution of the early moulting pathway in Ecdysozoa

**DOI:** 10.1098/rsos.180888

**Published:** 2018-09-12

**Authors:** Isabell Schumann, Nathan Kenny, Jerome Hui, Lars Hering, Georg Mayer

**Affiliations:** 1 Department of Zoology, Institute of Biology, University of Kassel, Kassel, Germany; 2 Molecular Evolution and Animal Systematics, Institute of Biology, University of Leipzig, Leipzig, Germany; 3 School of Life Sciences, Simon F.S. Li Marine Science Laboratory, Center of Soybean Research, State Key Laboratory of Agrobiotechnology, The Chinese University of Hong Kong, Shatin, Hong Kong, People's Republic of China

**Keywords:** ecdysis, ecdysone, 20-hydroxyecdysone, Halloween genes, Onychophora, Tardigrada

## Abstract

Moulting is a characteristic feature of Ecdysozoa—the clade of moulting animals that includes the hyperdiverse arthropods and less speciose groups, such as onychophorans, tardigrades and nematodes. Moulting has been best analysed in arthropods, specifically in insects and crustaceans, in which a complex neuroendocrine system acts at the genomic level and initiates the transcription of genes responsible for moulting. The key moulting hormones, ecdysone and 20-hydroxyecdysone, are subsequently synthesized from cholesterol ingested with food. Their biosynthesis is regulated by the Rieske-domain protein Neverland and cytochrome P450 enzymes encoded by the so-called ‘Halloween’ genes. Ecdysone is then released into the haemolymph and modified into 20-hydroxyecdysone, which binds to the nuclear receptor EcR/USP and initiates transcription of the Early genes. As little is known about the moulting pathway of other ecdysozoans, we examined the occurrence of genes involved in ecdysteroid biosynthesis and the early moulting cascade across ecdysozoan subgroups. Genomic and transcriptomic searches revealed no Halloween genes in cycloneuralians, whereas only *shadow* (*CYP315A1*) is present in onychophorans and tardigrades, suggesting that the Halloween genes evolved stepwise in panarthropods. These findings imply that the genes which were responsible for the ecdysteroid biosynthesis in the last common ancestor of Ecdysozoa are currently unknown.

## 1. Introduction

**Figure 1.**
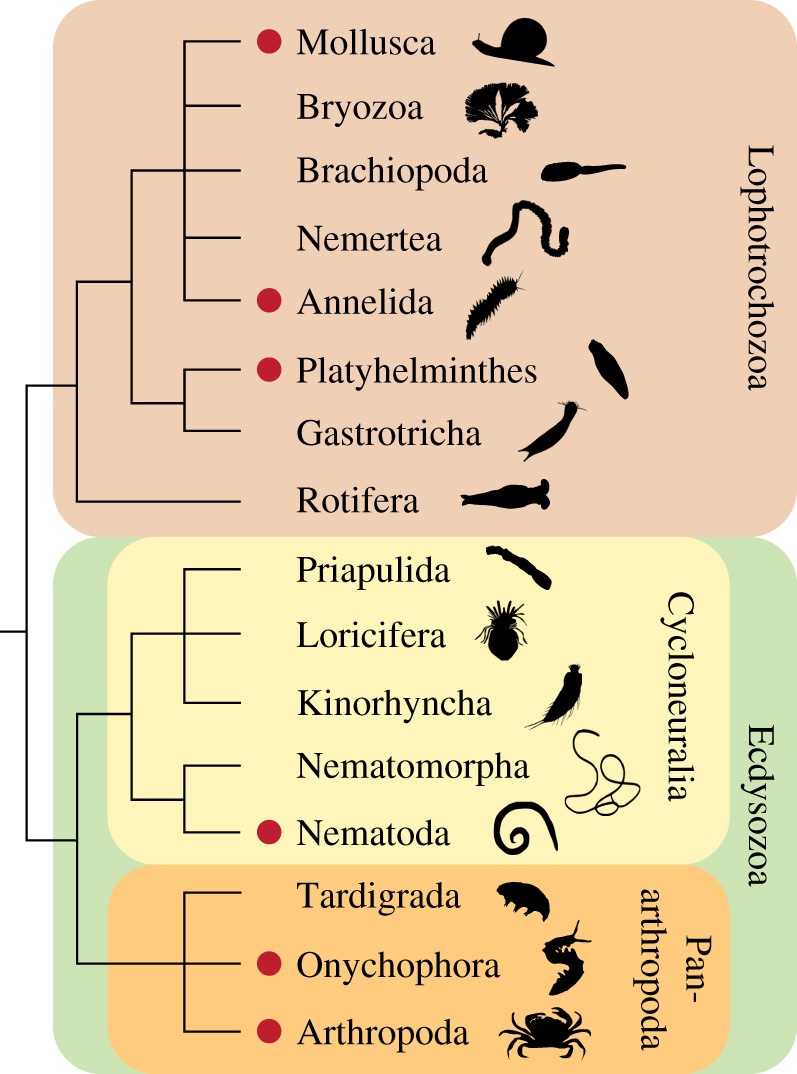
Identified occurrence of 20-hydroxyecdysone in protostomes. Note that 20-hydroxyecdysone (indicated by red dots) is not only found in ecdysozoans but also in other protostomes, including molluscs, annelids and platyhelminths. Phylogeny of protostomes modified from [1,2]. Note that cycloneuralians might not be monophyletic [1]. Animal silhouettes, except for those for Onychophora and Tardigrada, courtesy of PhyloPic (www.phylopic.org).

**Figure 2.**
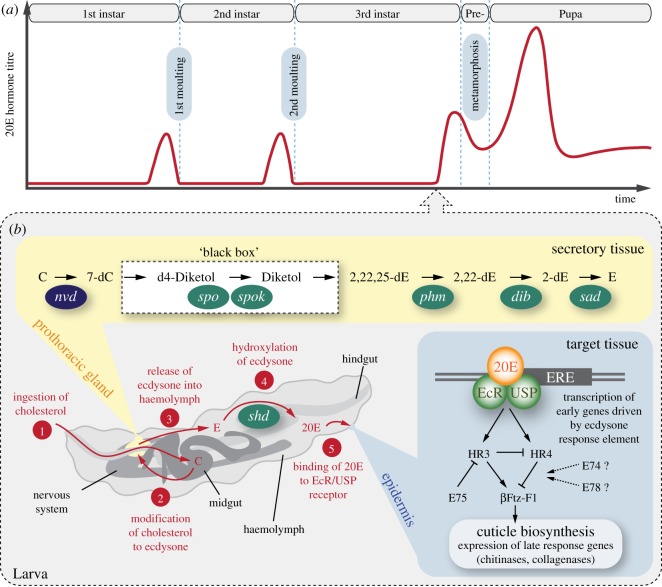
Hormonal titre and early moulting pathway in the fruit fly *Drosophila melanogaster.* Modified from [12,13]. (*a*) Changes of 20-hydroxyecdysone titre during development. Note that the titre increases shortly before each moult and metamorphosis. (*b*) Ecdysteroid biosynthesis and early moulting pathway in a 3rd instar larva (time point indicated by arrow in (*a*)). The numbers 1–5 indicate major steps of biosynthesis and binding to ecdysteroid receptor. Ingestion of dietary cholesterol is followed by ecdysone biosynthesis in secretory tissue (prothoracic gland), which is controlled by enzymes encoded by the Rieske-domain gene *neverland* (*nvd*), and the Halloween genes *spook* (*spo*), *spookier* (*spok*), *phantom* (*phm*), *disembodied* (*dib*) and *shadow* (*sad*). Ecdysone is then released into the haemolymph and oxidized to 20-hydroxyecdysone by an oxidase encoded by *shade* (*shd*). Binding of 20-hydroxyecdysone to the receptor complex EcR/USP in target tissue (epidermis) activates transcription of the Early genes (*E74*, *E75*, *E78*, *HR3*, *HR4* and *βFtz-F1*), which is followed by cuticle biosynthesis. 2-dE, 2-deoxyecdysone; 2,22-dE, ketotriol; 2,22,25-dE, ketodiol; 7-dC, 7-dehydrocholesterol; 20E, 20-hydroxyecdysone; C, cholesterol; E, ecdysone; *E74*, *ecdysone-inducible gene 74; E75, ecdysone-inducible gene 75; E78, ecdysone-inducible gene 78; HR3, hormone receptor 3; HR4, hormone receptor 4; βFtz-F1, beta Fushi-tarazu transcription factor 1;* EcR, ecdysone receptor; ERE, ecdysone response element; USP, Ultraspiracle.

Ecdysozoa is the sister group of Lophotrochozoa within protostomes and includes cycloneuralians (nematodes, priapulids, kinorhynchs and allies), and panarthropods (tardigrades, onychophorans and arthropods; figure 1). Although the phylogenetic relationships of ecdysozoans remain contentious (in particular, the validity of Cycloneuralia is under debate), the monophyly of the entire clade is supported by most molecular analyses and the process of moulting or ecdysis, which is considered an autapomorphy of this clade [1,3–6]. Ecdysis is essential for growth in these animals, as their body is typically covered with an inelastic cuticle or exoskeleton, which has to be shed and replaced periodically by larger covering. During this complex and strictly coordinated neuroendocrine process, precise timing of gene expression and physiological responses is essential for building the new cuticle or exoskeleton [7,8]. Hormones such as ecdysteroids have been demonstrated to be responsible for the control and regulation of moulting in arthropods [5,9]. Specifically, ecdysone (E) and 20-hydroxyecdysone (20E) have been identified as key players of ecdysis in insects [10] and crustaceans (crustecdysone *sensu* Hampshire & Horn [11]; figure 2*a,b*). These hormones are synthesized from the precursor sterol cholesterol, which is ingested with food, transported via a lipoprotein from the midgut to a secretory tissue and converted subsequently into the final moulting hormone 20E [14] (figure 2*b*).

In well-studied insects, such as the fruit fly *Drosophila melanogaster* and the tobacco hornworm *Manduca sexta*, the initial step in the biosynthesis of ecdysteroids is the dehydrogenation of cholesterol to 7-dehydrocholesterol, which takes place in the prothoracic gland and is catalysed by the Rieske oxygenase Neverland (Nvd) [15–17] (figure 2*b*). The subsequent hydroxylation reactions at different carbon atoms are catalysed by cytochrome P450 monooxygenases encoded by the Halloween genes *spook* (*spo*, *CYP307A1*), *phantom* (*phm*, *CYP306A1*), *disembodied* (*dib*, *CYP302A1*) and *shadow* (*sad*, *CYP315A1*). In *D. melanogaster*, two additional Halloween genes—*spookier* (*spok*, *CYP307A2*) and *spookiest* (*CYP307B1*)—have been identified with a putative hydroxylation function as part of the ‘Black box’ in the ecdysteroid pathway [18–20] (figure 2*b*). These multiple hydroxylation reactions in the secretory tissue produce ecdysone. This is released into the haemolymph and modified further to 20E by a monooxygenase encoded by the Halloween gene *shade* (*shd*, *CYP314A1*) in a species-specific target area such as the haemolymph, fat body or midgut [21,22]. The active moulting hormone 20E then binds to a high-affinity nuclear receptor complex that consists of two dimerization partners, Ecdysone Receptor (EcR) and the retinoid X receptor homologue Ultraspiracle (USP) [23–25]. By attaching to an ecdysone response element of the DNA, the 20E/EcR/USP complex initiates a hierarchical transcription cascade of the Early genes (at least six in *D. melanogaster*, including *E74*, *E75*, *E78*, *HR3*, *HR4* and *βFtz-F1*), which leads to the final biosynthesis of the new cuticle by the epidermis regulated by enzymes (e.g. chitinases and collagenases) encoded by the Late response genes [26–29] (figure 2*b*).

The molecular control of moulting is well understood in insects and crustaceans (e.g. [30–32]). However, little is known about this process in the remaining ecdysozoan subgroups, including the closest arthropod relatives, the onychophorans (velvet worms) and tardigrades (water bears) [1,12,33]. While in the onychophoran *Euperipatoides leuckartii* both key moulting hormones, E and 20E, have been identified using radioimmunoassay and high-performance liquid chromatography [34], corresponding data are unavailable from tardigrades, probably due to their minute body size (figure 1). To our knowledge, the occurrence of ecdysteroids has not been investigated in cycloneuralian taxa other than nematodes. Although ecdysteroids have been detected in the parasitic nematodes *Dirofilaria immitis* and *Ascaris suum* [35,36], neither E nor 20E have been identified in free-living nematodes, including the ‘model’ species *Caenorhabditis elegans* [37,38]. This would suggest a different pathway for the regulation of moulting in the free-living species of nematodes at least. Dafachronic acid, another sterol-derived hormone, has, for example, been identified as a moulting hormone in *C. elegans* [39].

Interestingly, the ecdysteroids E and 20E have also been detected outside the ecdysozoan clade, for example, in the cestode *Moniezia expansa*, the molluscs *Lymnaea stagnalis* and *Helix pomatia*, and the leech *Hirudo medicinalis* [40–44] (figure 1). Together with the observation that leeches periodically shed their cuticle [43,45,46], this raises the question of whether or not the ecdysteroid pathway is conserved among protostomes, and whether ecdysone-induced moulting predates the origin of Ecdysozoa.

To clarify this question, we explored the distribution of the major gene components of the early moulting pathway in insects across the Bilateria. In particular, we focused on the occurrence of the Halloween genes among ecdysozoans by including the sequenced genomes and transcriptomes of a priapulid, two tardigrade and one onychophoran species in our analyses (table 1). After identifying the relevant sequences, we performed phylogenetic analyses of the three major families of moulting genes, including those encoding a Rieske-domain protein, cytochrome P450 monooxygenases and nuclear hormone receptors. Our results contribute to a better understanding of the evolution of genes of the early moulting pathway in panarthropods. They also reveal substantial gaps in our knowledge of the ancestral moulting system of ecdysozoans.

**Table 1.** Ecdysozoan species, the genomes and/or transcriptomes of which were searched for homologues of genes of the moulting pathway.

taxon	species	genome	transcriptome	references
Scalidophora, Priapulida	*Priapulus caudatus*	✓		AXZU00000000.2 http://genome.wustl.edu/
Nematoda, Rhabditida	*Caenorhabditis elegans*	✓		[47]
Tardigrada, Eutardigrada	*Hypsibius exemplaris*	✓		[48]
			✓	[49]
	*Ramazzottius varieornatus*	✓		[50]
Onychophora, Peripatopsidae	*Euperipatoides rowelli*	✓		ftp://ftp.hgsc.bcm.edu/I5K-pilot/Velvet_worm/
			✓	[51]
Arthropoda, Chelicerata	*Ixodes scapularis*	✓		[52]
	*Tetranychus urticae*	✓		[53]
	*Stegodyphus mimisarum*	✓		[54]
Arthropoda, Myriapoda	*Strigamia maritima*	✓		[55]

## 2. Material and methods

### 2.1. Specimens, library preparation, sequencing and transcriptome assembly

Specimens of the onychophoran species *Euperipatoides rowelli* Reid, 1996 (Peripatopsidae) were collected from decaying logs in the Tallaganda State Forest (New South Wales, Australia, 35°26′ S, 149°33′ E, 954 m above sea level) in October 2011. The animals were kept in plastic jars with perforated lids at 18°C or as described previously [56,57] and fed with crickets (*Acheta domesticus*) every four weeks. Specimens of the eutardigrade species *Hypsibius exemplaris* Gąsiorek *et al.* [58] (Parachela, Hypsibiidae) were obtained commercially from Sciento (Manchester, UK) and kept in plastic Petri dishes filled with mineral water (Volvic, Danone Waters Deutschland GmbH, Frankfurt am Main, Germany) at 21°C and fed with unicellular algae (*Chlorococcum* sp.) as described previously [59–61]. This tardigrade species was commonly referred to as ‘*Hypsibius dujardini* (Doyère, 1840)’ in the literature (e.g. [48,49,62]), but Gąsiorek *et al.* [58] have described it as *Hypsibius exemplaris*. Library preparation, sequencing and assembly of transcriptomes for *E. rowelli* and *H. exemplaris* were performed as described previously [49,51].

### 2.2. Identification of genes and genome screening

To identify the sequences of putative genes from the early moulting pathway of various ecdysozoans (including *neverland*, Halloween genes, receptor genes, Early genes, and *CYP18A1*) (table 1), tBLASTn/BLASTp searches [63] of the NCBI GenBank database were conducted using an initial score cut-off of 80 and *E*-value of 1 × 10^−6^. Published sequences of the Halloween genes from different arthropods, including *Limulus polyphemus*, *Daphnia pulex* and *D. melanogaster*, were used as queries (see electronic supplementary material, file F1 and figures S1–S3 for accession numbers or references for all used sequences). When the target gene was not found by initial searches, further searches at a more permissive score of 60 and higher *E*-value cut-offs were performed. Alongside these searches, the available genomes of different ecdysozoan species, including *Priapulus caudatus*, *C. elegans*, *H. exemplaris*, *Ramazzottius varieornatus*, *E. rowelli*, *Ixodes scapularis*, *Tetranychus urticae*, *Stegodyphus mimisarum* and *Strigamia maritima* were screened for candidate genes using Hmmer or BLAST searches either directly on the Ensembl database [64] or on their respective webservers (table 1). Reciprocal tBLASTn/BLASTP searches of candidate gene sequences on UniProt and NCBI databases were performed for initial confirmation of gene identity from the examined genomes (table 1). Candidate sequences from the onychophoran *E. rowelli* and the tardigrade *H. exemplaris* were translated using the standard metazoan coding table with the online sequence translation tool EMBOSS Transeq ([65], http://www.ebi.ac.uk/Tools/st/emboss_transeq) and CLC Sequence viewer (Qiagen, 2017). The domain structures of all sequences (Rieske-domain for *neverland*, P450 domain for the Halloween genes and *CYP18A1*, Zn-Finger motif and hormone receptor for the receptor genes and the Early genes, ETS-domain for *E74*) were analysed using the Pfam database 31.0 [66] and SMART [67]. We used the corresponding sequences of the ETS transcription factor family to clarify the orthology of the identified *E74* sequences (*ETS*, *TCF*, *PEA3* and *ERG sensu* Sharrocks *et al.* [68]).

### 2.3. Sequence alignment and phylogenetic analyses

Amino acid sequences of putative genes of the early moulting pathway (59 for *neverland*, 238 for the Halloween genes and *CYP18A1*, 173 for the nuclear hormone receptors and 58 for the ETS transcription factor family genes) were used for phylogenetic analyses. We generated dataset and domain sequence alignments for each gene family (257 amino acids in length for *neverland*, 1006 for the Halloween genes and *CYP18A1*, 665 for the nuclear hormone receptors and 83 for the ETS transcription factor family genes) using the online tool MAFFT version 7 [69] and the L-INS-I strategy (see electronic supplementary material, file F2 for all alignments). The Pthread-Version of RAxML v. 8.2.X [70] was used to infer the best maximum-likelihood tree to reveal the phylogenetic position of each candidate sequence by using a dataset-specific GTR substitution matrix (-m PROTGAMMAGTR). For each run, the best tree was obtained from 10 independent inferences. Bootstrap support values for all trees were calculated using the rapid bootstrapping algorithm implemented in RAxML from 1000 pseudoreplicates. Trees were visualized with iTol v. 4.0.3 [71] and edited with Illustrator CS5 (Adobe Systems, San Jose, CA, USA).

## 3. Results

### 3.1. Homologues of the Rieske-domain gene *neverland*

Our genomic/transcriptomic searches and phylogenetic analyses revealed homologues of *neverland* (*nvd*) in numerous bilaterians (table 2; see electronic supplementary material, figure S1 for phylogenetic analyses). We identified orthologues of *nvd* in all analysed arthropod species, including the chelicerate *I. scapularis*, the myriapod *S. maritima,* the crustacean *D. pulex* and the insect *D. melanogaster*. While genomic and transcriptomic data from Kinorhyncha, Loricifera and Nematomorpha are still absent from available databases, we confirmed the previously identified homologue of *nvd*, *daf-36* (cf. [17]), in the nematode *C. elegans*. By contrast, we found no *nvd* homologues in genomic and/or transcriptomic data from the onychophoran *E. rowelli*, the tardigrades *H. exemplaris* and *R. varieornatus*, and the priapulid *P. caudatus* (table 2; see electronic supplementary material, figure S1 for phylogenetic analyses).

**Table 2.** Identified homologs (✓) of candidate genes involved in the moulting process across major ecdysozoan taxa. Note that genomic or deep transcriptomic data are unavailable for Loricifera, Kinorhyncha, and Nematomorpha. Abbreviations: *βFtz-F1*, *beta fushi tarazu transcription factor 1*; *CYP18A1*, *cytochrome P450-18A1*; *dib*, *disembodied*; *EcR*, *ecdysone receptor*; *HR3*, *hormone receptor 3*; *HR4*, *hormone receptor 4*; *nvd*, *neverland*; *phm*, *phantom*; *sad*, *shadow*; *shd*, *shade*; *spo*, *spook*; *spok*, *spookier*; *USP/RXR*, *ultraspiracle/retinoid X receptor*.

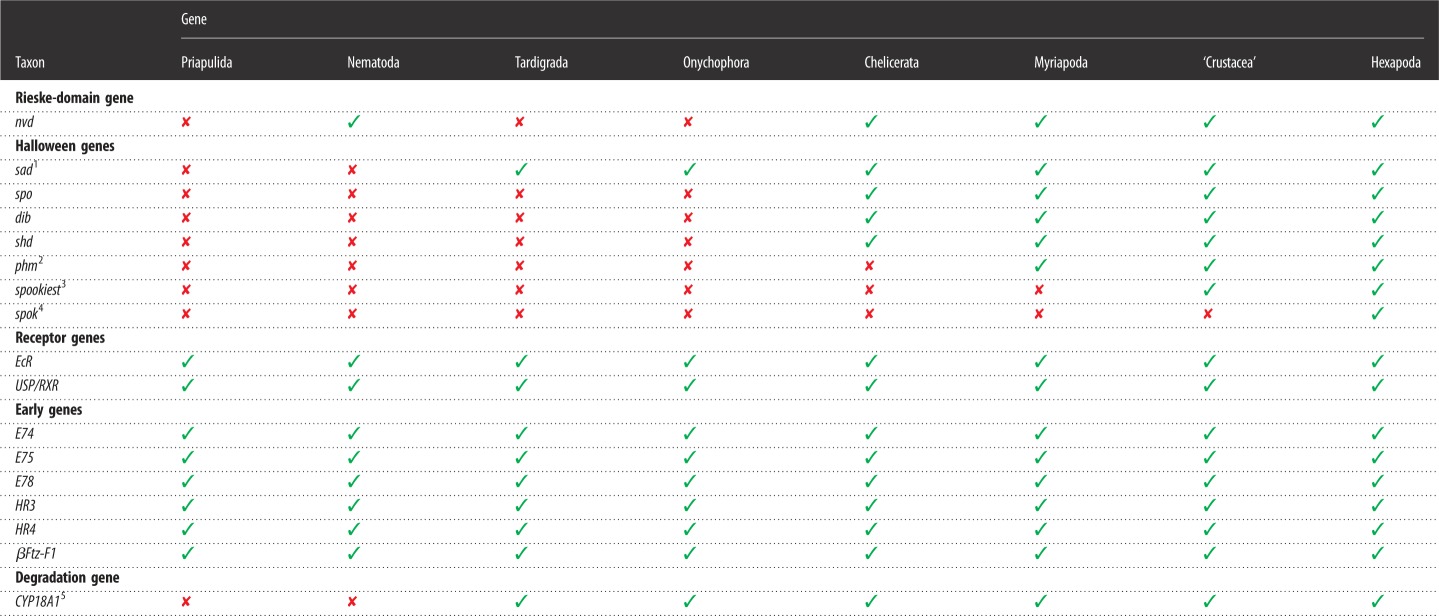

^1^Three copies of *sad* are present in the onychophoran *Euperipatoides rowelli* (Peripatopsidae).

^2^Homologues of *phm* are missing in the genomes of the chelicerates *Stegodyphus mimosarum*, *Ixodes scapularis* and *Tetranychus urticae*.

^3^*spookiest* might have been lost in the cladocerans *Daphnia pulex* and *D. magna*, as its homologues are missing in their genomes.

^4^Homologues of *spook* seem to be present only in drosophilids.

^5^*CYP18A1* is responsible for the degradation of 20-hydroxyecdysone in insects.

### 3.2. Cytochrome P450 family genes

**Figure 3.**
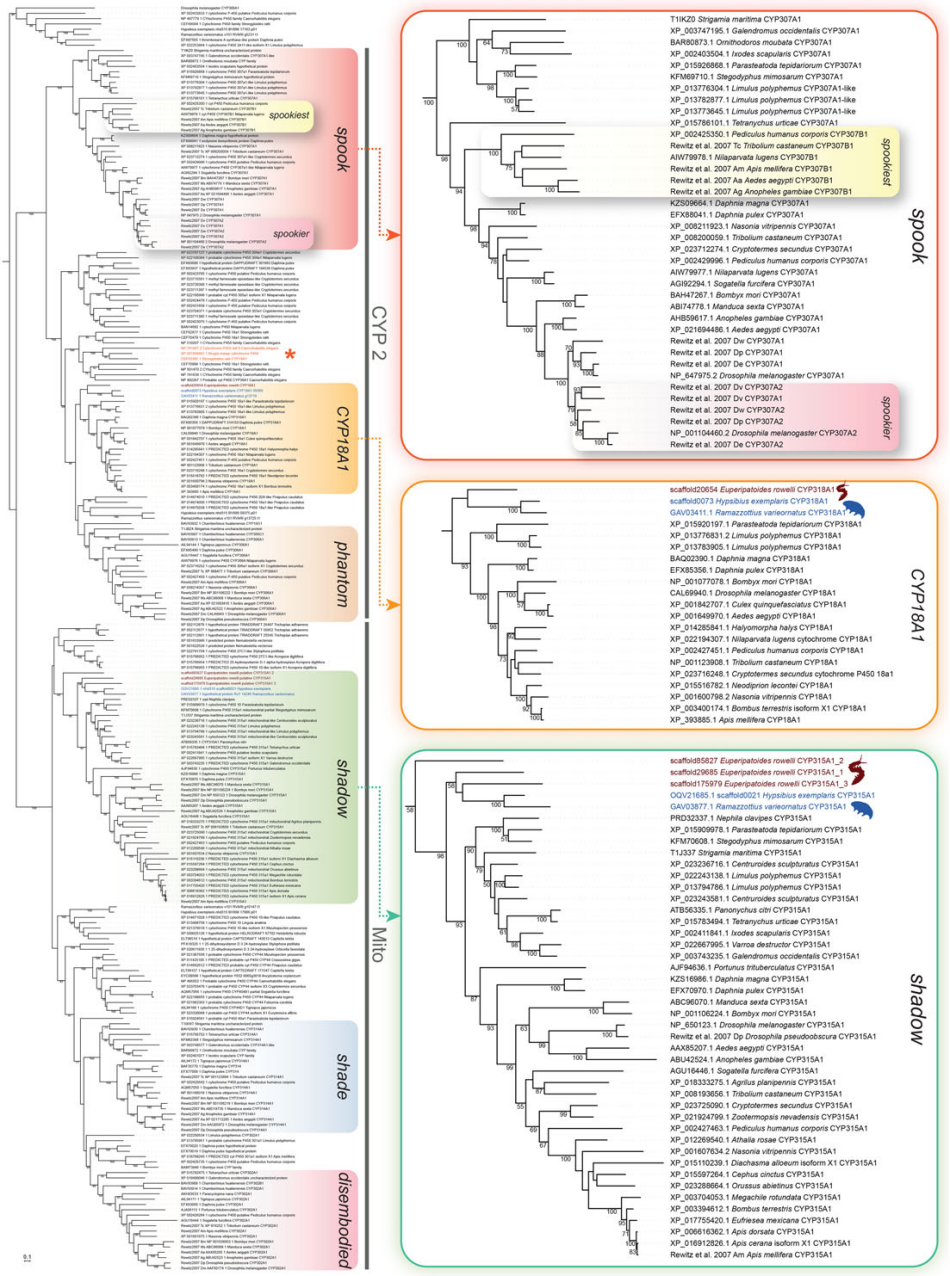
Phylogenetic relationship of the cytochrome P450 family genes. The tree is based on a maximum-likelihood analysis of 238 P450 domain sequences of the mitochondrial (Mito) and cytochrome P450 family 2 (CYP2) from different ecdysozoans (see electronic supplementary material, file F1 for identified sequences and accession numbers, and figure S2 for uncondensed tree). Numbers at nodes indicate bootstrap support values greater than 50% obtained from 1000 pseudoreplicates. Note that the homologues of the Halloween genes *disembodied* (*dib*), *phantom* (*phm*), *shade* (*shd*) and *spook* (*spo*) are missing in onychophorans and tardigrades. Single homologues of *CYP18A1* are present in the onychophoran *Euperipatoides rowelli* (highlighted in brown) and the tardigrades *Hypsibius exemplaris* and *Ramazzottius varieornatus* (both highlighted in blue). Three copies of *shadow* (*sad*, *CYP315A1*) are present in the onychophoran *E. rowelli* (highlighted in brown) and one copy in the tardigrades *H. exemplaris* and *R. varieornatus* (highlighted in blue). Note further that the nematode *daf-9* sequences occur within a clade of other nematode cytochrome P450 family genes, indicating that *daf-9* might not be an orthologue of *CYP18A1* (orange asterisk).

To clarify the orthology of the Halloween genes, we analysed the phylogeny of selected cytochrome P450 family genes (figure 3). Our screening of genomic and transcriptomic data from the onychophoran *E. rowelli* and the tardigrades *H. exemplaris* and *R. varieornatus* revealed only orthologues of the Halloween gene *shadow* (*sad*, *CYP315A1*) in these three panarthropod species (figure 3 and table 2). While the two tardigrade species show single homologues of *sad*, we identified three transcripts of *sad* in the onychophoran *E. rowelli* (figure 3). The tardigrade and onychophoran homologues form a clade together with the corresponding sequences from arthropods (figure 3). Besides the homologues of *sad*, our results revealed four additional Halloween genes (*spo*, *dib*, *shd* and *phm*) in the myriapods *S. maritima* and *Chamberlinius hualienensis*, the crustaceans *D. pulex* and *Daphnia magna*, and several insect species, whereas *phm* is missing in the chelicerates *S. mimosarum*, *I. scapularis* and *T. urticae* (table 2; see electronic supplementary material, figure S2 for uncondensed phylogenetic analyses)*.* In contrast to onychophorans, tardigrades and arthropods, the genomic data from nematodes and the priapulid *P. caudatus* exhibit no Halloween genes.

Our searches further revealed putative orthologues of the cytochrome P450 gene *CYP18A1* at least in all major subgroups of panarthropods studied (figure 3 and table 2). Interestingly, the *daf-9* sequences from the nematodes *C. elegans*, *Brugia malayi* and *Strongyloides ratti* (indicated by an orange asterisk in figure 3) fall into an assemblage of other nematode cytochrome P450 family genes, which cluster as the sister group to a clade comprising *CYP18A1*, *phm* and other P450 sequences (figure 3). We did not find homologues of *CYP18A1* in the priapulid *P. caudatus* (figure 3 and table 2).

### 3.3. Nuclear receptor and ETS transcription factor genes

**Figure 4.**
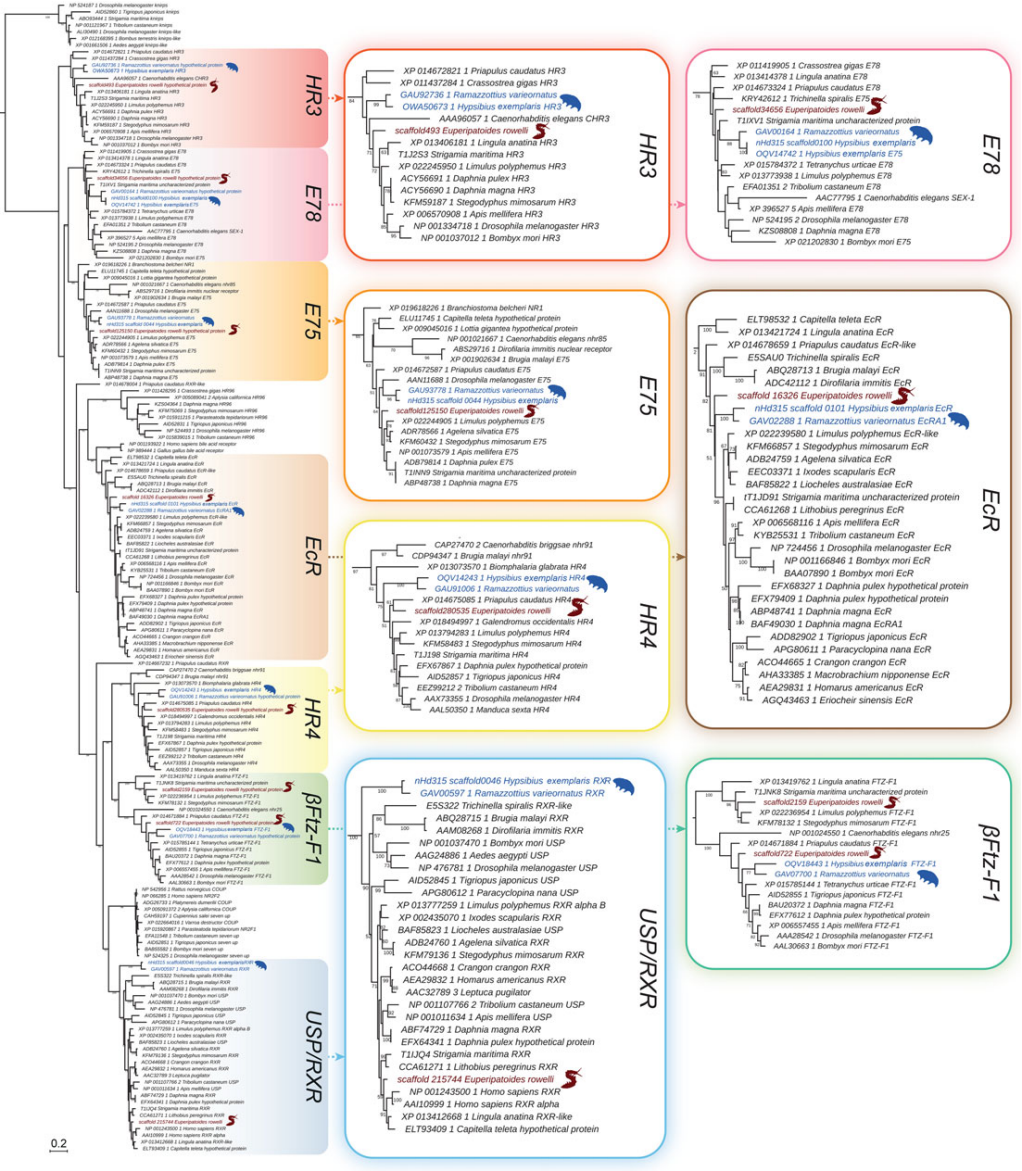
Phylogenetic relationship of the nuclear hormone receptor genes. The tree is based on a maximum-likelihood analysis of 173 sequences from different bilaterian species (see electronic supplementary material; file F1 for accession numbers and electronic supplementary material, figure S3 for uncondensed tree). Numbers at nodes indicate bootstrap support values greater than 50% obtained from 1000 pseudoreplicates. Note that the homologues of *ecdysone receptor* (*EcR*), *beta fushi tarazu transcription factor 1* (*βFtz-F1*), *hormone receptor 3* (*HR3*), *hormone receptor 4* (*HR4*), *ecdysone-inducible genes E75* and *E78*, and *ultraspiracle/retinoid X receptor* (*USP/RXR*) are present in the onychophoran *Euperipatoides rowelli* (highlighted in brown) and the tardigrades *Hypsibius exemplaris* and *Ramazzottius varieornatus* (both highlighted in blue).

**Figure 5.**
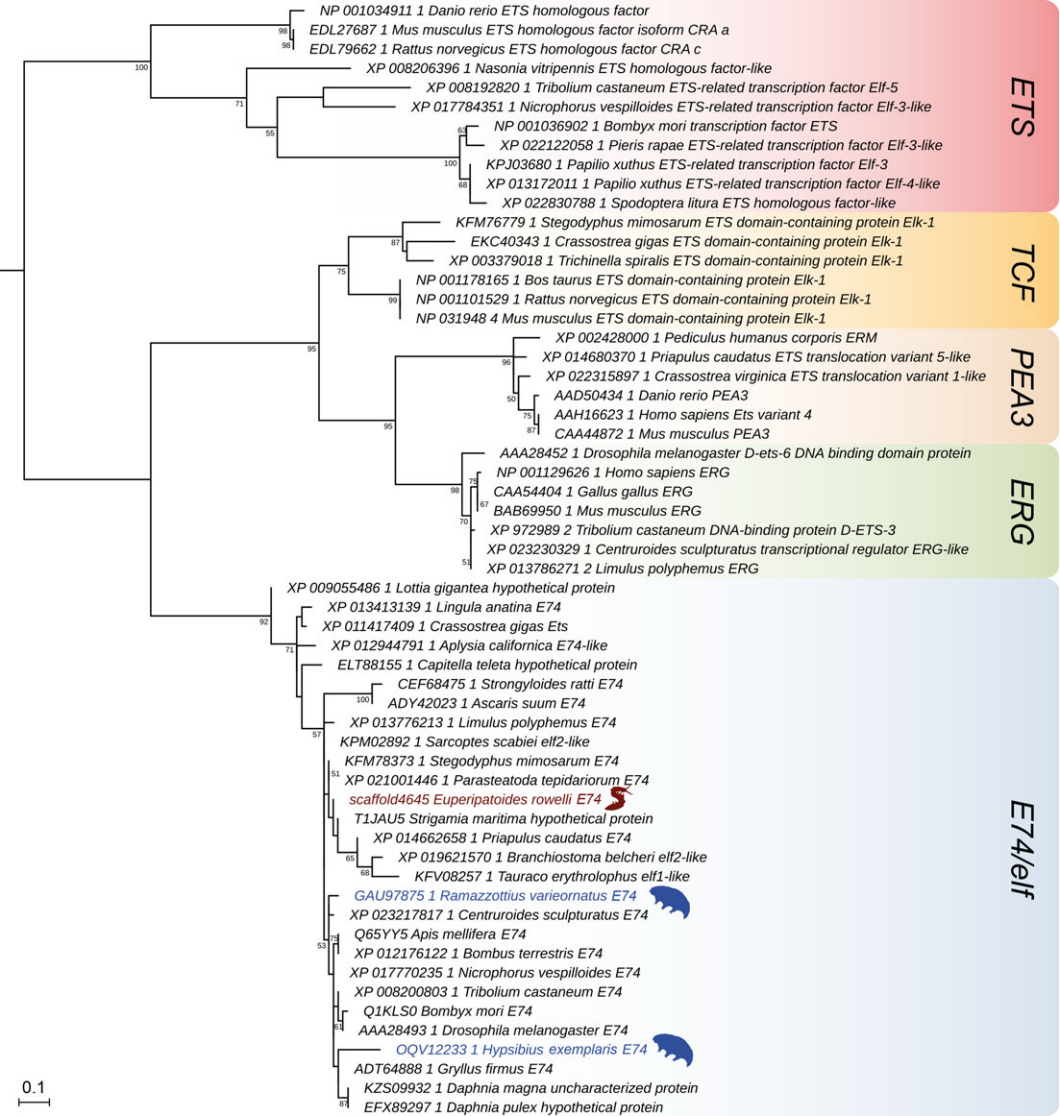
Phylogenetic relationship of ETS family genes including *E74*. The tree is based on a maximum-likelihood analysis of 58 sequences from different bilaterians (see electronic supplementary material, file F1 for identified sequences and accession numbers). Note the presence of *E74* homologues in the onychophoran *Euperipatoides rowelli* (highlighted in brown) and the tardigrades *Hypsibius exemplaris* and *Ramazzottius varieornatus* (both highlighted in blue).

We further searched the available databases for homologues of the nuclear hormone receptor genes *EcR*, *ultraspiracle/retinoid X receptor* (*USP/RXR*), and the Early genes *E75*, *E78*, *HR3*, *HR4* and *βFtz-F1* (figure 4 and table 2). We used *knirps* and related sequences, which are part of the nuclear hormone receptor family, from several arthropods as an outgroup. Besides representative ecdysozoan sequences, we included *EcR* and *USP/RXR* homologues from the annelid *Capitella teleta*, the brachiopod *Lingula anatina* and the vertebrates *Gallus gallus* and *Homo sapiens* in our sampling. We identified homologues of *EcR* and *USP/RXR*, which encode the two dimerization partners of the moulting receptor complex in arthropods, in all studied ecdysozoan subgroups (figure 4 and table 2). Interestingly, we found no orthologues of *EcR* and *USP* in the genome of the free-living nematode *C. elegans*, although the corresponding sequences are present in the parasitic nematodes *Trichinella spiralis*, *Dirofilaria immitis* and *Brugia malayi*. Among the Early genes, which also belong to the nuclear hormone receptor family, we identified *E75*, *E78*, *HR3*, *HR4* and *βFtz-F1* in all studied ecdysozoans (figure 4 and table 2). The same holds true for the homologues of the ecdysone-inducible Early gene, *E74*, which belongs to the ETS transcription factor family (figure 5 and table 2).

## 4. Discussion

### 4.1. Loss of *neverland* homologues in Tardigrada, Onychophora and Priapulida

The Rieske-domain gene *nvd* encodes an oxygenase, which in insects acts upstream of the ecdysteroid biosynthesis pathway and dehydrogenates cholesterol to 7-dehydrocholesterol [15,17] (cf. figure 2*b*). However, Nvd is also known to play a more general role in the transport and metabolism of cholesterol [72]. Based on our results, we propose that a homologue of *nvd* might have existed in the last common ancestor of bilaterians, in which it might have played an ancient role in the metabolism of steroids, as these hormones are essential for development, growth and homeostasis in both protostomes and deuterostomes [17]. Surprisingly, we did not find homologues of *nvd* in the onychophoran *E. rowelli*, the tardigrades *H. exemplaris* and *R. varieornatu*s and the priapulid *P. caudatus*, suggesting independent losses of this gene in the corresponding lineages. Owing to these losses, the enzyme responsible for the transformation of cholesterol to 7-dehydrocholesterol in these three animal groups is currently unknown.

### 4.2. Stepwise evolution of Halloween genes in Panarthropoda

The Halloween genes encode hydroxylases that, at least in crustaceans and insects, convert 7-dehydrocholesterol into ecdysone and 20-hydroxyecdysone [73–75]. Mutations of the Halloween genes in the fruit fly *D. melanogaster* result in ecdysteroid deficiency and lead to cuticle deformations and embryonic lethality [76,77]. Despite their essential role in the fruit fly, our results revealed no homologues of Halloween genes in cycloneuralians, suggesting that these animals might use an alternative pathway for the biosynthesis of ecdysteroids, or they might even use another type of moulting hormones. The free-living nematode *C. elegans* indeed seems to use dafachronic acid instead of 20E as the main moulting hormone [38,78], whereas the parasitic nematodes *A. suum* and *B. malayi* do show a molecular response to ecdysone [79–81]. Hence, the question arises as to whether the dafachronic acid pathway or the ecdysteroid pathway was responsible for the biosynthesis of moulting hormones in the last common ancestor of Cycloneuralia, provided this group is monophyletic [1]. Genome sequencing and, in particular, experimental studies in additional cycloneuralian taxa, such as priapulids, kinorhynchs, loriciferans and nematomorphs, might help to clarify this question.

**Figure 6.**
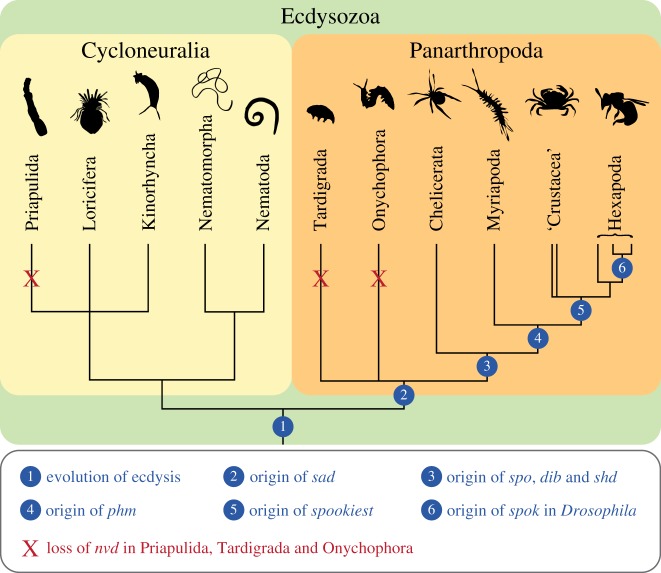
Scenario describing stepwise evolution of genes of the early moulting pathway in Ecdysozoa. Note that the last common ancestor of Panarthropoda possessed only the Halloween gene *shadow* (*sad*) and that the remaining Halloween genes, including *spook* (*spo*), *disembodied* (*dib*), *shade* (*shd*), *phantom* (*phm*), *spookiest* and *spookier* (*spok*), evolved stepwise in the arthropods.

Our results further show that the repertoire of Halloween genes varies considerably among arthropods. For example, the canonical 20E biosynthesis pathway of *D. melanogaster* exhibits six Halloween genes: *spo*, *spok*, *phm*, *dib*, *sad* and *shd* (cf. figure 2*b*), whereas most other panarthropods possess distinct sets of Halloween genes. While *spookiest* is missing in *Drosophila* species, this gene does occur in other insects and some crustaceans, suggesting that *spookiest* was present in the last common ancestor of Pancrustacea but was lost in some lineages, including the drosophilids and the cladoceran crustacean *Daphnia* (figure 6). Moreover, our phylogenetic analyses revealed that *spok* is a drosophilid in-paralogue of *spo*, thus confirming that *spok* might have evolved by gene duplication in the drosophilid lineage [18,75]. Although homologues of *spo*, *phm*, *dib*, *sad* and *shd* are present in myriapods, crustaceans and insects, *phm* is apparently missing in chelicerates (as speculated in Qu *et al.* [82]). The lack of this gene implies that *phm* was either lost in chelicerates or evolved in mandibulates (figure 6). Finally, *sad* is the only Halloween gene we identified in the onychophoran *E. rowelli* and the tardigrades *H. exemplaris* and *R. varieornatus*. While single orthologues of *sad* are present in tardigrades, the onychophoran transcriptome and genome databases exhibit three copies of this gene, suggesting that there might have been two duplication events in the onychophoran lineage or an onychophoran subclade.

In summary, based on our findings we propose that the most plausible scenario is a stepwise evolution of Halloween genes in panarthropods (figure 6). While only *sad* was present in the last common ancestor of Panarthropoda—a condition which has been retained in extant onychophorans and tardigrades—the genes *spo*, *dib* and *shd* might have arisen in the arthropod lineage, followed by the evolution of *phm* in mandibulates. The Halloween gene *spookiest* might have originated in the pancrustacean lineage, while *spok* evolved in drosophilids. These findings suggest that the biosynthesis pathway of ecdysteroids in tardigrades, onychophorans and chelicerates must be different from the canonical pathway of *D. melanogaster*. Owing to the lack of *phm*, the conversion of 2,22,25-dE to 2,22-dE (cf. figure 2*b*) might be accomplished by another enzyme in chelicerates, which is unknown. Alternatively, the lack of *phm* in these animals might be due to the use of ponasterone A (25-deoxy-20E) instead of 20E as a moulting hormone [82]. The situation is even less clear in onychophorans and tardigrades. Even if their only Halloween gene *sad* does participate in the conversion of 2-dE to E (cf. figure 2*b*)—a function which still has to be demonstrated experimentally—the remaining members of the ecdysteroid pathway in these animals are unknown. Hence, virtually nothing is known about the components of the ecdysteroid pathway and the molecular mechanisms of moulting in the last common ancestor of Panarthropoda.

### 4.3. Evolution of the EcR/USP complex and the Early genes

Another important process in the ecdysteroid pathway of insects is binding of 20E to the heterodimeric receptor complex EcR/USP, which activates the hierarchical transcription cascade of the Early genes [25–27] (figure 2*b*). The individual subunits of this receptor complex, which is also essential for reproduction and embryogenesis [83–85], are encoded by the *EcR* and *USP/RXR* genes. While *EcR* encodes three isoforms of the EcR subunit in *D. melanogaster* and two in the tobacco hornworm *M. sexta* [86,87], our analyses revealed single transcripts of *EcR* in the onychophoran *E. rowelli*, and the tardigrades *H. exemplaris* and *R. varieornatus*. We further identified single homologues of *USP/RXR*—the gene encoding the dimerization partner of EcR—in the onychophoran and the two tardigrade species. The occurrence of 20E and homologues of *EcR* and *USP/RXR* in several ecdysozoan taxa, including priapulids and the parasitic nematodes, suggests that the transcription of the Early genes might have been triggered by binding of 20E (or a related molecule) to the EcR/USP complex in the last common ancestor of Ecdysozoa. This ancient role of EcR/USP in steroid binding might have been inherited from the last common ancestor of protostomes, as genes encoding both receptor subunits are also found in various other protostomes outside the ecdysozoan clade.

After steroid binding, the 20E/EcR/USP complex binds to an ecdysone response element in the promoter region and initiates the expression of the Early genes that encode transcription factors controlling the time and specificity of moulting in insects [29,88–90]. Our genomic and transcriptomic analyses revealed homologues of six Early genes in the onychophoran *E. rowelli*, and the tardigrades *H. exemplaris* and *R. varieornatus*: five of which were classified as the nuclear hormone receptor genes (*E75*, *E78*, *HR3*, *HR4* and *βFtz-F1*), and one belonging to the ETS family of transcription factors (*E74*). Although the function of these genes has been well analysed in insects [90–93], only little is known about their potential role in moulting in other ecdysozoans. Studies on the nematode *C. elegans* have demonstrated that beyond their roles in development and egg-laying, at least some of the Early genes are involved in moulting [94,95]. Together with our finding of a complete set of Early genes in all studied ecdysozoan subgroups and other protostomes, such as the brachiopod *L. anatina* and the annelid *C. teleta*, this suggests that the Early genes might have been present in the last common ancestor of protostomes and were recruited for moulting in Ecdysozoa, irrespective of whether their transcription was initiated by 20E or another steroid hormone.

### 4.4. Evolution of the ecdysteroid-inactivating enzyme cytochrome P450-18A1 in panarthropods

In addition to ecdysone oxidase, the cytochrome P450 protein CYP18A1 has been characterized as an ecdysteroid-inactivating enzyme, which plays an important role in the development and metamorphosis of insects [96]*.* In the fruit fly *D. melanogaster* and the moths *B. mori* and *M. sexta*, the 26-hydroxylase encoded by *CYP18A1* affects developmental processes by inactivating 20E and degrading it to 20,26-dihydroxyecdysone through the addition of an OH group to the 26th carbon of the hormone molecule [96,97]. Our study revealed homologues of *CYP18A1* in all major panarthropod subgroups, including the onychophoran *E. rowelli*, in which CYP18A1 might play a similar role in 20E degradation as this ecdysteroid has been demonstrated in the closely related species *E. leuckartii* [25]. By contrast, we found no unambiguous homologues of *CYP18A1* in available genomes from nematodes and the priapulid *P. caudatus*, suggesting that this gene might have been recruited for degradation of 20E (or a related hormone) in the last common ancestor of Panarthropoda.

## 5. Conclusion

To clarify whether the early moulting pathway of the fruit fly *D. melanogaster* is a conserved feature of Ecdysozoa, we analysed candidate genes of this pathway in various bilaterians. While the homologues of genes encoding the heterodimer receptor complex EcR/USP and the Early genes are present in all major ecdysozoan subgroups, to our surprise we found only a few genes of the canonical ecdysteroid pathway in these taxa. Although our results revealed no homologues of the Rieske-domain gene *nvd* in onychophorans, tardigrades and priapulids, this gene might have been present in the last common ancestor of Ecdysozoa and was most likely lost in these three ecdysozoan subgroups (figure 6). Hence, it is unclear whether the dehydrogenation of cholesterol to 7-dehydrocholesterol occurs in these animals and, if so, which enzyme accomplishes this dehydrogenation. Similarly, we found homologues of *CYP18A1*—a gene belonging to the degradation pathway of 20E—only in panarthropods; it is, therefore, unclear which gene was responsible for the degradation of ecdysteroids in the last common ancestor of Ecdysozoa.

Another unexpected result of our study is the finding that most likely none of the Halloween genes were part of the ecdysteroid pathway in the last common ancestor of Ecdysozoa, and that only the Halloween gene *sad* was present in the last common ancestor of Panarthropoda (figure 6). Although functional assays would be required to clarify whether or not this gene is involved in the hydroxylation of ecdysone to 2-deoxyecdysone in onychophorans and tardigrades, our results clearly indicate that *sad* was the first Halloween gene to evolve in the panarthropod lineage, whereas the remaining Halloween genes might have originated subsequently in arthropods by gene duplications within the cytochrome P450 superfamily.

Taken together, these results suggest that most of the key players responsible for the conversion of cholesterol to 20E (or a related hormone) in the last common ancestor of Ecdysozoa are currently unknown. This is astonishing, given that ecdysis—a process which is believed to be governed by the ecdysteroid hormones—is regarded as the most prominent autapomorphy of this clade [3–5]. Future studies should, therefore, focus on aspects of the endocrine control of this process in understudied ecdysozoan subgroups, to gain a full understanding of this vital process in this important clade.

## Supplementary Material

Figure S1

## Supplementary Material

Figure S2

## Supplementary Material

Figure S3

## Supplementary Material

File F1

## Supplementary Material

File F2

## Data accessibility

All data are available in the electronic supplementary material.
