# Supplementary figures and images for: Halloween genes in panarthropods and the evolution of the early moulting pathway in Ecdysozoa
Source: R Soc Open Sci. 2018 Sep 12;5(9):180888. doi: 10.1098/rsos.180888 (PMC6170570; doi:10.1098/rsos.180888)

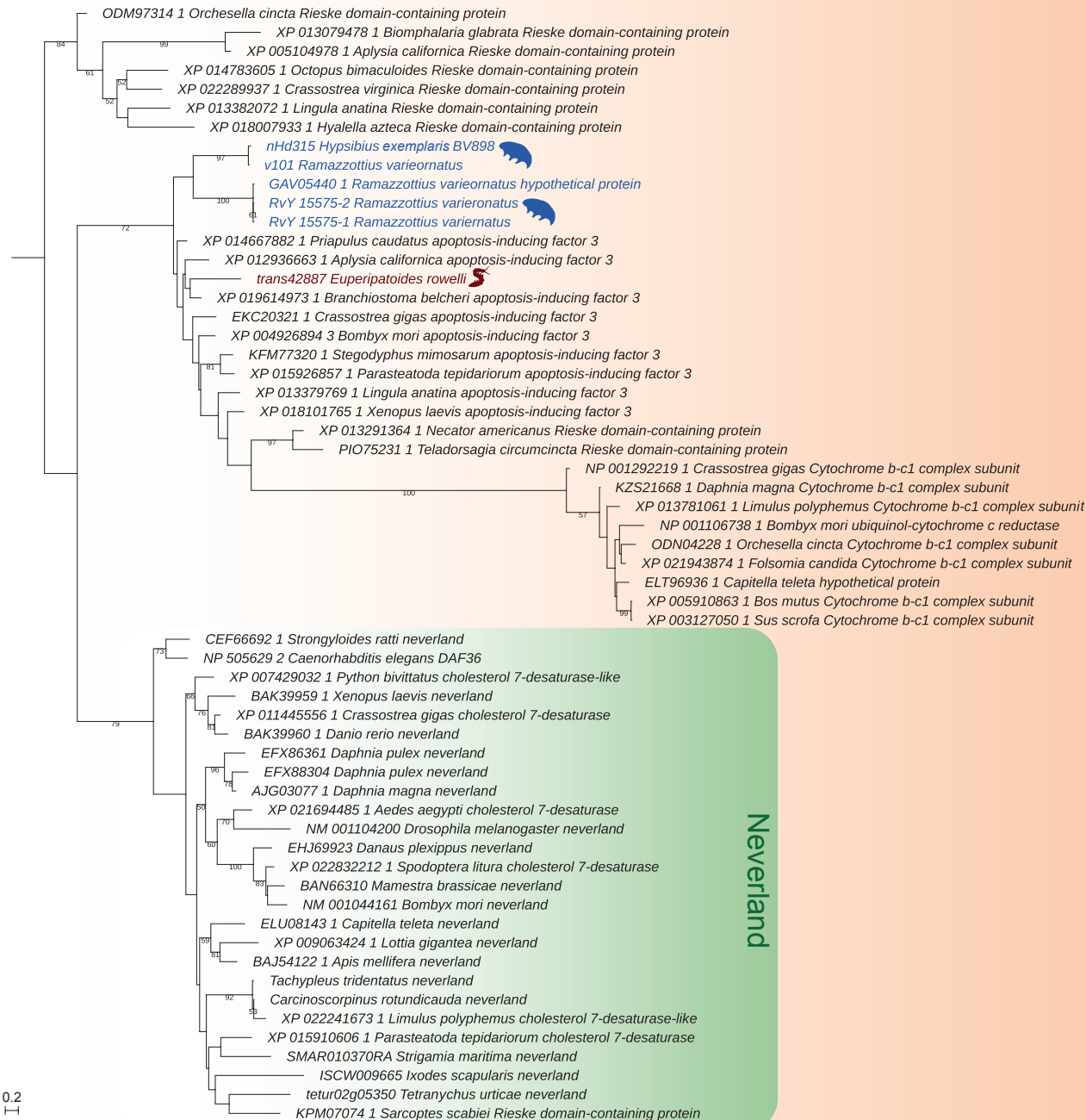

Supplement: Figure S1 [file rsos180888supp1.pdf]

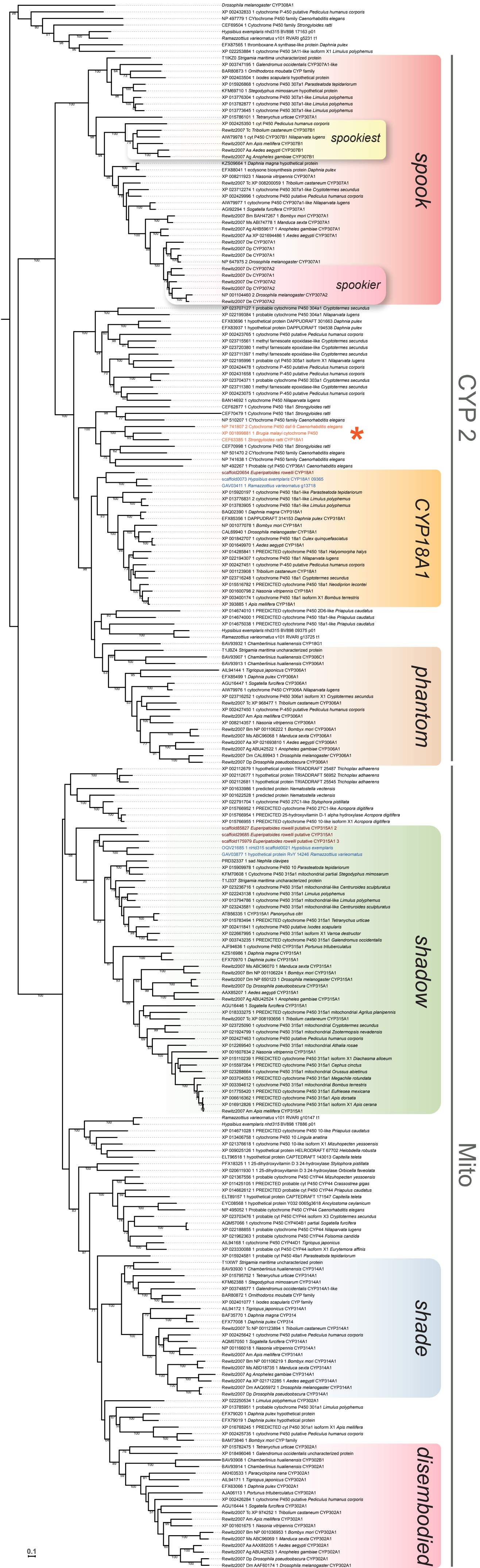

Supplement: Figure S2 [file rsos180888supp2.pdf]

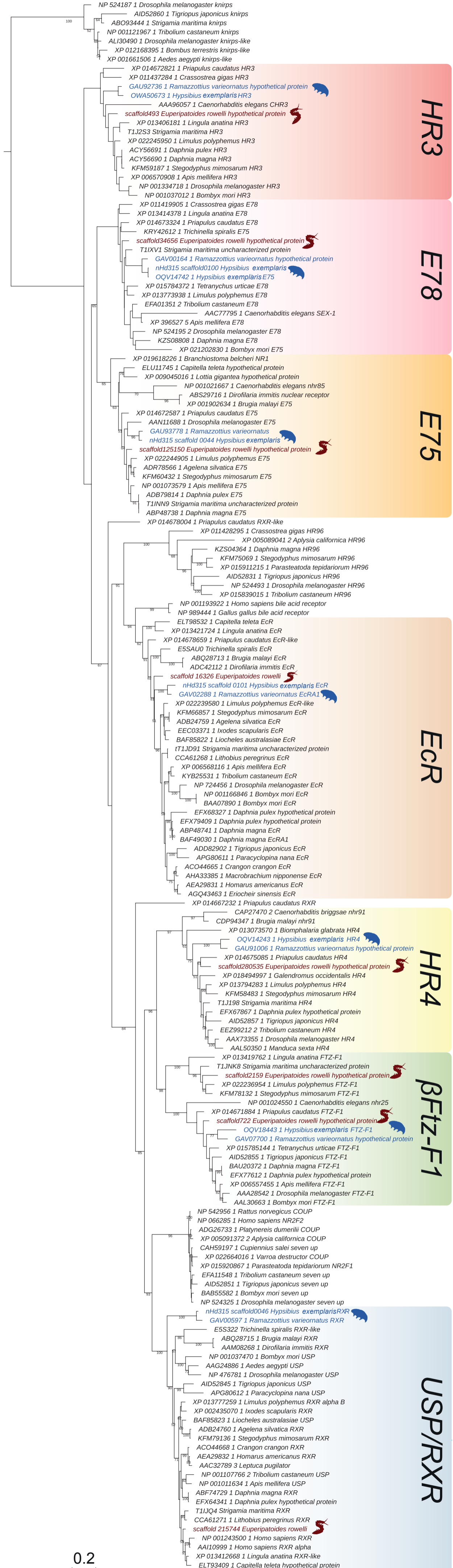

Supplement: Figure S3 [file rsos180888supp3.pdf]
